# Advancing primary care education: Lessons from the United Kingdom for Japan

**DOI:** 10.1002/jgf2.70058

**Published:** 2025-08-29

**Authors:** Lauren Glover, Takashi Watari, Tomoko Miyoshi, Hitomi Kataoka

**Affiliations:** ^1^ University of Birmingham Medical School Birmingham UK; ^2^ Integrated Clinical Education Center Kyoto University Hospital Kyoto Japan; ^3^ General Medicine Center Shimane University Hospital Izumo Shimane Japan; ^4^ Center for Medical Education and Internationalization Kyoto University Kyoto Japan

Primary care is a core component of most healthcare systems. Patients often present with symptoms that can be investigated and treated by primary care clinicians without requiring further specialist input. Although the way primary care is provided differs across countries, it serves various purposes, including providing continuity of care, coordinating with secondary care services, and offering a patient‐centered approach to encourage individuals to take control of their health.

Despite its global importance, the clinical education system in Japan places limited focus on primary care. Undergraduate education primarily focuses on specialists and secondary care. Although structured training programs have recently been established in general practice and family medicine, physicians often enter primary care roles without undergoing formal retraining. Given that Japan's rapidly aging population will lead to an increase in patients with complex multimorbidities and long‐term conditions, physicians in Japan must be equipped with the knowledge to treat them through an effective primary care education system.

There are several differences between primary care education in the United Kingdom and Japan (Table 1). Primary care in the United Kingdom is mainly delivered through the specialty of General Practice. The United Kingdom places a strong emphasis on GP training as a core part of undergraduate education, with the General Medical Council (GMC), the UK's medical regulatory body, advising that all students should be placed in General Practice.^1^ Medical students in the United Kingdom typically spend longer studying General Practice than students in Japan. Research suggests that across all UK medical schools, students spent a median of 53 full days either in teaching sessions or on placement at GP surgeries.^2^ In contrast, the Japanese medical curriculum remains largely focused on specialist care, with the median duration of community‐based clinical training estimated at approximately 12 days to 4 weeks.^3^ Therefore, UK graduates typically gain more experience in primary care than their Japanese counterparts, potentially increasing their understanding of the GP specialty when leaving medical school.

**TABLE 1:** The table summarizes key differences in primary care education between Japan and the United Kingdom at undergraduate and postgraduate levels.

Feature of training	United Kingdom	Japan
Undergraduate medical education	In the undergraduate medical curriculum, primary care is firmly embedded across all 5 years, accounting for approximately 13% of total teaching time.^7^ Students frequently engage in supervised clinical consultations in primary care, allowing for the early development of patient‐facing skills under the guidance of licensed general practitioners	The Japanese medical curriculum remains primarily oriented toward specialist care, with the median duration of community‐based clinical training estimated at approximately 12 days to 4 weeks.^3^ Although medical education is shifting to a more participatory approach, Japanese medical students rarely engage in direct patient consultations before graduation, as most clinical experiences are observational or limited to supervised settings
Postgraduate training before specialty	The UK foundation program, undertaken after medical school, follows a formalized curriculum that often includes a community‐based placement for 4 months, which is in some cases spent in general practice	Approximately 4 weeks spent in community‐based medicine over 2 years, with significant variation in the content and quality of these rotations. Postgraduate training in large hospitals in Japan often provides limited opportunities for active engagement in primary care
Training pathways	Three years of postgraduate training program completed after foundation training, including time spent in relevant hospital specialties and usually 18–24 months in GP posts.^4^ A relatively high proportion of doctors enter this training pathway compared to Japan, with approximately 32% of doctors entering the GP specialty training program after completing the UK foundation program in 2019^6^	A formalized training pathway for GPs was established in 2018 by the Japanese Board of General Medicine (JBGM) in which doctors undertake General Medicine training; however, the uptake remains low. In 2022, only 250 doctors began GP training, comprising 2.65% of the total number of residents.^3^ Once doctors have completed the JBGM program, they can complete further specialist training in family medicine with the Japan Primary Care Association (JPCA)
Certification	Managed by the Royal College of GPs (MRCGP), multiple assessments are required to be completed to become a registered GP through the MRCGP, including an Applied Knowledge Test, Simulated Consultation Assessment, and Workplace‐Based Assessment.^4^ In addition to this, an annual review of competence progression is undertaken	Physicians can obtain certification through the JPCA Family Medicine Program. To become certified, the JPCA has trainees complete a multiple‐choice examination, evaluating 12 areas of practice. Additionally, a written detailed case report is completed.^5^ However, such certification is not a mandatory requirement to practice as a primary care physician in Japan^8^
Continuing education and assessment	GPs must undertake an annual portfolio‐based appraisal as part of revalidation every 5 years	The JPCA has an established renewal process for doctors who have completed the GP training pathway, including detailed case reports and an examination.^5^ However, the renewal requirements for doctors working in primary care who have not completed the JPCA GP training pathway are perhaps less clear
Curriculum and scope of practice	GPs have a clearly defined curriculum outlining their role in caring for patients with conditions relating to all medical specialties,^4^ including key knowledge and skills all GPs should have. GPs have scope to develop their interests further and can have extended roles in several defined specialties to help meet the needs of their population	There are 12 areas of knowledge expected that GPs meet set by the JPCA^5^ in guidance for examination. However, given that many doctors working in primary care have not completed this training pathway, the scope of practice is often narrower and ambiguously defined, with significant variability based on individual clinician discretion. Terms such as “family medicine,” “hospital medicine,” and “general internal medicine” are inconsistently applied, reflecting the lack of a standardized framework^9^

After graduating, doctors in the United Kingdom undertake 2 years of foundation training, which is equivalent to 2 years of junior residency in Japan. During these 2 years, all doctors should undertake community placement, meaning that many of them experience 4 months of work in General Practice. Upon completion, doctors in the United Kingdom choosing to specialize in GP complete three further years of specialty training. This involves the time spent in the relevant hospital departments and approximately 18–24 months in GP posts.^4^ Following the completion of this program, doctors must pass the Royal College of General Practitioner (RCGP) exams to practice independently, including a multiple‐choice paper, simulated consultation assessment, and workplace‐based assessments. This assessment is guided by a curriculum that ensures that all fully qualified GPs have acquired certain skills and knowledge to standardize care and promote safe practices. Once fully qualified, GPs continue to be assessed through revalidation every 5 years, which involves completing an annual portfolio. Therefore, primary care is well integrated into both undergraduate and postgraduate training in the United Kingdom, with a well‐defined training pathway for becoming a primary care practitioner.

In Japan, to become a GP, after completing residency, doctors undertake 3 years of the General Medicine specialist training program. Established by the Japanese Board of General Medicine (JBGM) in 2018, it includes training in various specialties, including internal medicine, rural medicine, and ambulatory care. If doctors then choose to work in clinical settings instead of in a hospital, they can undertake additional training offered by the Japan Primary Care Association (JPCA) to become a Family Medicine specialist,^5^ largely equivalent to the UK GP role. To become certified by the JPCA, trainees complete a multiple‐choice exam, evaluating 12 defined areas of practice, and a written case report is completed.^5^ However, unlike in the United Kingdom, completion of this training pathway is not mandatory to work in primary care settings. The uptake of the JBGM pathway in 2022 was only 2.65% of new resident doctors,^3^ a proportion unlikely to meet the growing need for primary care specialists in Japan. This contrasts substantially with the United Kingdom, where approximately 32% of doctors enter GP specialty training after completing the foundation program,^6^ meaning the UK primary care workforce benefits from being more standardized.

The UK primary care education approach has several advantages in terms of primary care outcomes. Comprehensive undergraduate and postgraduate education in primary care ensures that doctors qualifying in the United Kingdom acquire knowledge on delivering holistic, patient‐centered care, and have a broad understanding of the various social determinants of health. This is key to providing comprehensive care for patients with chronic conditions and practicing preventative medicine. This also allows UK students to develop an interest in becoming generalists from early in their medical careers, which helps sustain the GP workforce. As mentioned previously, a greater proportion of doctors in the United Kingdom than in Japan enter the formalized GP training pathway after completing their residency. This compulsory training pathway benefits patient safety by ensuring that despite variations between individual practitioners in terms of interests and specific expertise, all doctors working unsupervised in primary care are formally qualified as GPs and have met a set of predefined competencies that have been set out in the RCGP curriculum to obtain this certification.^4^

Japan's current primary care approach, in which specialists deliver care to patients with specific symptoms, has several advantages over the UK system. Patients can quickly consult experts regarding their condition. Furthermore, this system provides doctors in Japan with more flexibility in tailoring their individual careers to suit them, as they can switch from hospital careers to community‐based careers more easily. Although doctors can undertake training activities as part of moving to a primary care career, they do not have to complete the mandatory GP training program. This contrasts with the United Kingdom, where although previous clinical experience can reduce the time required for training, formalized GP training must be completed before working in primary care, regardless of the doctor's career stage. In Japan, many doctors working in primary care come from specialist backgrounds without completing formalized GP training, although many have met the requirements set by the JPCA's Board Certification or Generalist Residency Program. However, this implies significantly greater variation in the training backgrounds of doctors working in primary care in Japan than in the United Kingdom, where all doctors working as GPs must complete the same training pathway based on a set curriculum. This could imply less standardization in the specific competencies of different doctors working in primary care in Japan and could be a disadvantage to patient safety due to less consistency in the required competencies across primary care providers.

Overall, it appears that the UK's primary care education system is well established and more standardized than is currently the case in Japan, owing to a comprehensive undergraduate training system overseen by the GMC, with more time spent studying primary care. Additionally, the much higher uptake of the national standardized primary care training program in doctors' post‐residency in the United Kingdom means that doctors working in primary care have less variability in their training backgrounds than in Japan. The undergraduate curriculum in Japan now emphasizes a participatory approach, including increasing clinical clerkship hours and OSCE assessments as part of training. It is important for medical schools in Japan to increase their focus on primary care during undergraduate education to encourage more doctors to work in this field and supply all graduates with the skills needed to practice holistically and in a patient‐centered manner. The primary care setting is ideal for enabling students to practice practical skills such as medical interviewing and clinical examinations through seeing undifferentiated patients under supervision. Therefore, introducing more clinical placements in this area would improve knowledge of primary care and benefit practical skills and knowledge relevant to various specialist areas. This would hopefully help develop interest in primary care careers at an earlier stage to increase the proportion of Japanese residents undertaking the JBGM and JPCA GP training pathways, as research from the UK indicates that undergraduate GP experience makes students more likely to pursue careers as GPs later on.^2^ Therefore, increasing undergraduate primary care education is crucial in Japan, as the need for comprehensively trained primary care doctors is growing in conjunction with the aging population.

## AUTHOR CONTRIBUTIONS

All authors have access to the information utilized and participated in the preparation of this manuscript.

## FUNDING INFORMATION

None.

## CONFLICT OF INTEREST STATEMENT

Dr. Takashi Watari and Dr. Hitomi Kataoka are Editorial Board members of JGFM Journal and co‐authors of this article. To minimize bias, they were excluded from all editorial decision making related to the acceptance of this article for publication.

## ETHICS STATEMENT

Ethics approval statement: None.

Patient consent statement: None.

Clinical trial registration: None.

## CONSENT FOR PUBLICATION

All authors have provided consent for publication.

## Data Availability

The data that support the findings of this study are available from the corresponding author upon reasonable request.
